# Case of Injury Received in Dissection

**Published:** 1827-02

**Authors:** John Shaw

**Affiliations:** Surgeon to the Middlesex Hospital.


### INJURY FROM DISSECTION.

*Case of Injury received in Dissection.* By JOHN SHAW, Surgeon to the MIDDLESEX HOSPITAL.

THE following history relates to a subject so interesting to all members of the medical profession, that it is unnecessary to make any apology for laying it before your readers. The first part is nearly in the words of the patient, a practitioner in town.

"When I awoke, (on Sunday morning, 7th January,) the fingers of my left hand felt stiff, attended with an acute and hot smarting pain, as if they had been burnt: the pain extended over the back of the hand, and up the arm to the axilla, and was particularly severe above the inside of the elbow, where the gland was already swollen and painful to the touch. I had headache; dull, heavy coldness of the feet and the other hand. On getting up, I felt stiff and sore in all my limbs; the muscles at the back of my neck were peculiarly so, and an uneasiness extended along the left side to the foot and ankle. On going to wash, I perceived a small pustule on the last joint of the middle finger of the left hand. It was very painful, and the finger appeared inflamed and swollen. I now recollected that I had, about half-past one the day before, grazed it with the eye-end of the needle, while sewing up the body of a person who died of peritonitis.

"Feeling myself exceedingly nervous and depressed, at half-past nine, I took two table-spoonfuls of brandy in a cup of coffee. Immediately after this, I went to see a patient; but the pain continued to extend over the left pectoral muscle, and deep under the axilla; the general uneasiness and coldness also increased, and I became more languid and dejected. While walking through Portman-square, I was seized with a fit of laughter, over which I had no control; and, when in the patient's house, I could scarcely restrain myself from crying. I got home a little after ten; and now I felt an oppression in my breathing, with a constriction in my throat, which produced difficulty of swallowing; the skin of the face felt tightened as if starched, and the jaws were stiff. The deadly coldness increasing, at a quarter to eleven I took three-spoonfuls of brandy.

"Dr. Ferguson, who had been present at the examination of the body, now called: he did not object to the use of the brandy, but, as he was just setting off to the country, desired me to send for Mr. Shaw. In the mean time, the general coldness and depression increasing, I took more brandy, and so freely (as I have since learned) that I had taken three-quarters of a pint before a quarter-past twelve. A medical friend then visited me, and advised me not to take any more brandy. He prescribed four grains of calomel, and seven leeches to the wrist, which was now much inflamed." Such was Mr. ——'s account.

Having been engaged, in another part of town, in examining the body of a patient who had died of peritonitis, (which, when I saw this gentleman, gave me some occasion of alarm,) I did not reach South-street until a quarter past one. I found the patient reclining on two chairs, with his arm lying on a table: the wrist and hand were of a livid red; a similar patch was on the inside of the arm, above the elbow; the finger was a little swollen, but the wound was scarcely perceptible. His countenance was very extraordinary, his expression being that of a mixture of hysterical alarm and intoxication. From his general appearance I thought he was delirious, but, when my name was mentioned, he said "I am glad to see you, Mr. Shaw," (I had never seen him before;) "but I am a dead man; I feel that nothing can save me. They have put leeches to my hand, but all is useless: I was poisoned in opening a body." He then burst into an hysterical laugh. When told of the quantity of brandy he had taken, I supposed that the peculiarity of his appearance was produced by it; but I have since learned from Dr. Ferguson, who saw him when he had taken only two table-spoonfuls, that he was then nearly in the same state.

With much difficulty I got him up stairs to bed: on lying down, he said, "I shall never rise from this bed." I immediately gave him a bolus of eight grains of calomel and six of colocynth, with two grains of opium, and wrapped his arm, from the fingers to the shoulder, in a towel steeped in a lotion made of four ounces of tincture of opium, two drachms of the liquor acetatis plumbi, and sixteen ounces of water. I remained with him a short time, and, finding him more composed, I ordered that laudanum should be freely poured over the cloth, so as to keep it quite wet; and that an infusion of an ounce of powdered opium in a pint of boiling water should be immediately prepared. I returned in about an hour: learning that he was more composed, I did not go up to him, but desired that a linseed poultice should be made with the infusion of opium, and applied to his hand and wrist; and the lotion of laudanum and lead to be constantly applied until I visited him again.

In the evening, I requested my friend Mr. Griffiths, of Bentinck-street, (who, while house-surgeon of St. George's Hospital, had an attack somewhat similar,) to accompany me. We found our patient quite composed, but dejected and low. His hand and arm were not so much inflamed as in the middle of the day. We ordered ten grains of the Dover's powders, with four of calomel, to be taken immediately; and after it a draught of an ounce of camphor mixture, eight grains of carbonate of ammonia, fifteen drops of laudanum, and half an ounce of mucilage. This was to be repeated every four hours, if awake. The lotion and poultice to be continued. He was to take a little gruel.

On calling about ten in the morning, we were not a little surprised to find that he had gone to the neighbourhood of

Burton Crescent, to attend a patient in labour. We were told that he had only retained about two and a half of the draughts, having vomited up the rest. I returned at three, expecting to find him again in bed, but I found him in his parlour, and he appeared to be so well that I recommended him only to continue the lotion and poultice, and to take some strong beef-tea.

At seven in the evening, Dr. Ferguson, Mr. Griffiths, and I met. Mr. ——— was now quite composed, could give a connected report of his state of feeling previously to my seeing him. He had not even head-ache; which he observed was extraordinary, as when, on any former occasion, he had taken even a glass of spirits, he had suffered even excessively from it. The hand and wrist, instead of being tense and livid, were white and corrugated, and without pain; but there was still a blush of redness above the elbow. His bowels had been freely opened. We now prescribed ten grains of the Dover's powder and four of calomel, with five grains of the Pilula Sapon. c. Opio; and desired him to take a glass of brandy in his gruel, and to continue the lotion and poultice.

On calling next morning, I again found that he was out. I saw him at three, continuing better: he had not taken the calomel, as his mouth felt sore. On Thursday he called, and saw me at the hospital: the inflammation of the arm had nearly subsided, but I advised him to keep his arm in a sling for a few days.

I did not see him again until to-day (the 24th): he has been quite well for some days; no abscess formed.

Notwithstanding Mr. ———'s rapid recovery, I think, when the symptoms he laboured under are compared with those fatal cases detailed by Dr. Duncan and others, and especially with the account given by Dr. A. T. Thomson of his own sufferings, there can be little doubt that this was one of the serious cases which ensue from wounds received during dissection. The symptoms could not in this instance be ascribed to thecal inflammation, for there never was more than a pustule. I fear that at present too much importance is attached to the effect of the local irritation. I am well aware of the alarming symptoms that sometimes ensue from the confinement of matter, but the prevailing theories on this question are, in my opinion, replete with danger.

From the history of this case, I think we are justified in concluding—That the symptoms resembled those following the bite of a venomous animal, more than those consequent on thecal inflammation, and that they proceeded from a cause independent of mere local inflammation;—that, immediately on the commencement of such symptoms, stimuli are not injurious;—that it is not necessary to bleed in such cases;*—that the combination of calomel, opium, and stimuli, even of the most powerful kind, seems to be useful; —that the lotion of acetate of lead and opium is a powerful auxiliary to the general method of treatment.†

The plan of treatment which I have proposed differs very much from that generally advised. I was led to recommend stimuli from remembering the relief I myself experienced when suffering from a wound received in the dissecting-room. Dr. Thomson seems to have proceeded on the same grounds; and we have, in the preceding history, an extraordinary example of the extent to which the stimulating plan may be carried. I would not recommend a similar course to be pursued in another case; but it seems to prove that, in the first state, the disease is not inflammatory.

Perhaps your readers will allow me to make the following quotation from a former communication to your Journal in May 1825. After describing the nature and treatment of wounds received in the dissecting-room, I have stated that—

"The other class of cases is more serious, as it constitutes an affection to which the practitioner is more liable than the student, and one that is attended with immediate danger to life.

"All who have been in the habit of attending to morbid anatomy, must admit that there is more danger from a wound received during the examination of the body of a person who has died from any form of inflammation of the peritoneum, than of any other disease. So convinced am I of this, that I have for many years inculcated the necessity of being particularly careful when examining a body, even though it is not putrid, where death has been caused by puerperal fever, the operation for hernia, or any form of peritonitis, or, I may add, even of pleuritis.

"If, five or six hours after examining such a body, pain is felt in the finger, and a small pimple or a blush of red is found at the part, immediate attention should be paid to it. If the case proceeds in the usual manner, there is a darting pain up the arm, which seems to fix more particularly in the shoulder or side of the chest. Within fourteen hours, the patient is very ill; he suffers a great deal of pain, and is anxious and alarmed. Red lines may generally be perceived running from

* It must not be forgotten that, in almost all the cases which have terminated fatally, the patients have been bled either by the lancet or leeches. Mr. —— did not lose two ounces of blood: I took off the leeches immediately on seeing him.

† This lotion is almost a specific in subduing the inflammation consequent on the wounds received in the dissecting-room: I have generally been able to prevent the formation of abscess. If Mr. ——'s case be compared with Dr. Thomson's, I think it will be admitted that the lotion prevented the formation of abscess.

the hand towards the axilla, but somtimes there are no marks on the arm, nor even on the finger. Indeed, the affection of the finger is occasionally so slight, that it is neglected, and the patient refers all his sufferings to the shoulder and chest.

"With regard to the treatment, the most important question seems to be, what is to be done during the first twenty-four or forty-eight hours? for, after this time, the peculiar character of the disease is in a great measure lost. The patient may fall into a low typhoid state, or he may labour under the symptoms of the erysipelas phlegmonoides; so that after a few days there may be a question as to whether he is to be stimulated and supported, or to be reduced by bleeding.

"Are we to bleed in the first instance? Is a man to be bled because he has a pulse at 140, headache, anxiety, and difficulty of breathing, and flushing of the face? Under almost any other circumstances, excepting in those I have mentioned, I believe no one would for a moment doubt the propriety of a copious bleeding, when such symptoms are present: but may not the pulse be raised by a morbid irritation,—the anxiety and flushing of the face proceed from terror,—the headache from the same cause,—and the difficulty of respiration be owing partly to the same, and to the morbid condition of the principal respiratory muscles? But we seldom have the face flushed; it is generally pale and haggard, with a cold perspiration on the brow; and the tongue has not the white dry surface, nor has the pulse the hard stridulous beat, that marks the inordinate action which requires to be subdued by blood-letting. Proceeding on these grounds, and on the conclusions I have drawn from the accounts given of the effects of bleeding in the cases that are recorded, I have objected to venesection. I have also objected to local bleeding by leeches; for the swelling and inflammation of the arm is of a character that does not appear to be benefited by such treatment; and although, from the cases recorded, we may conclude that local bleeding has been sometimes of advantage, still its good effects have not been sufficiently marked to induce me to run the risk of reducing in this way the vital energies of my patient."

As no bad effects resulted from the free use of brandy in the case I have detailed, and as depletion by bleeding has been tried in all the cases which have terminated fatally, I think I am justified in still giving the same opinion as to the way I should treat myself, were I to be affected in a similar manner.

"The moment the pain commenced, I would immerse my finger in equal parts of laudanum and Goulard's lotion; I

would take a smart dose of calomel and antimony, and, two hours afterwards, a large dose of laudanum. If the pain still continued, I would bathe my whole hand and fore-arm with tepid laudanum and Goulard,—take another opiate, perhaps with a little ammonia,—and even force myself to swallow cordials in the form of warm negus, &c."

---

